# Pros and cons of a wandering mind: a prospective study

**DOI:** 10.3389/fpsyg.2013.00524

**Published:** 2013-08-14

**Authors:** Cristina Ottaviani, Alessandro Couyoumdjian

**Affiliations:** ^1^IRCCS Santa Lucia FoundationRome, Italy; ^2^ENPlab, Department of Psychology, Sapienza University of RomeRome, Italy

**Keywords:** mind wandering, ecological momentary assessment, prospective study, sleep, heart rate, heart rate variability, somatization

## Abstract

Mind wandering (MW) has recently been associated with both adaptive (e.g., creativity enhancement) and maladaptive (e.g., mood worsening) consequences. This study aimed at investigating whether proneness to MW was prospectively associated with negative health outcomes. At time 0, 21 women, 19 men; mean age = 24.5 (4.9) underwent a 5-min baseline electrocardiogram (ECG), a 20-min laboratory tracking task with thought probes, and personality questionnaires. At time 1 (1 year follow-up), the same participants underwent a 24-h Ecological Momentary Assessment characterized by ambulatory ECG recording and electronic diaries. First, we examined if the likelihood of being a “mind wanderer” was associated with specific personality dispositions. Then, we tested if the occurrence of episodes of MW in the lab would be correlated with frequency of MW in daily life. Finally, multiple regression models were used to test if MW longitudinally acted as a risk factor for health, accounting for the effects of biobehavioral variables. Among dispositional traits, the frequency of MW episodes in daily life was inversely associated with the capacity of being mindful (i.e., aware of the present moment and non-judging). There was a positive correlation between frequency of MW in the lab and in daily life, suggesting that it is a stable disposition of the individual. When differentiated from perseverative cognition (i.e., rumination and worry), MW did not predict the presence of health risk factors 1 year later, however, a higher occurrence of episodes of MW was associated with short-term adverse consequences, such as increased 24-h heart rate (HR) on the same day and difficulty falling asleep the subsequent night. Present findings suggest that MW may be associated with short term “side effects” but argue against a long term dysfunctional view of this cognitive process.

## Introduction

Mind wandering (MW) has been defined as the default mode of operation of our brain (Mason et al., 2007), and it has been associated with maladaptive consequences for health (reviewed in Mooneyham and Schooler, 2013). Despite the pervasiveness of MW (almost 50% of our waking time in Killingsworth and Gilbert, 2010), little is known about its functionality. It has been hypothesized that MW plays a vital role in healthy cognition (Baars, 2010), and recent studies suggest adaptive functions that are served by MW. For example, MW appears to integrate past and present experiences for the purpose of future planning and simulation (i.e., autobiographical planning; Baird et al., 2011; Smallwood et al., 2011a). Consistent with this hypothesis, the MW experience is often future focused (Smallwood et al., 2009a; D'Argembeau et al., 2011), and oriented toward personal goal resolution (e.g., Baird et al., 2011; D'Argembeau et al., 2011; Smallwood et al., 2011a). Inspiration is another function that can be intuitively associated with MW, especially considering the well-known benefits of an incubation interval for creative thoughts. Thus, Baird et al. (2012) demonstrated that MW facilitates creative problem solving. A growing number of studies (e.g., Baird et al., 2011; Levinson et al., 2012) indicate that the capacity to mentally escape from the constraints of the present permits the management of personal goals (e.g., Smallwood and Schooler, 2006; Baumeister and Masicampo, 2010). Consistent with this view, Smallwood et al. (2013) demonstrated that MW is associated with reduced delay discounting, suggesting that MW allows cognition to be devoted to the consideration of personal objectives that extend beyond the current moment, becoming relevant for making choices that are beneficial over the long term. This seems to be true across cultures, as a recent ecological study of a Chinese population showed that MW helped participants to create and maintain an integrated, meaningful sense of self and to cope with upcoming events (Song and Wang, 2012). Among other functions, Gruberger et al. (2011) hypothesized that MW may serve as a learning and consolidation mechanism by augmenting the associative abilities of the brain, in a similar way to what happens when we sleep.

On the flip side, MW has been paradoxically associated with unconstructive consequences in terms of reduced attention and interference with performance on tasks that require substantial controlled processing (reviewed in Mooneyham and Schooler, 2013). A number of studies linked MW to poor performance in sustained attention tasks, such as vigilance tasks (Smallwood et al., 2004a; Allan Cheyne et al., 2009; Mrazek et al., 2012) or reading (Smallwood et al., 2008; Reichle et al., 2010; Smilek et al., 2010; Franklin et al., 2011; McVay and Kane, 2012). During reading, MW leads to slower reading times, longer average fixation duration, and absence of the word frequency effect on gaze duration (Foulsham et al., 2013), with a negative influence on the comprehension of difficult texts (Feng et al., 2013). Moreover, MW has been associated with worse performance on measures of fluid intelligence and working memory (Mrazek et al., 2012). Taken together, these impairments can lead to serious consequences that go from the more obvious scholastic failure (Smallwood et al., 2007a) to traffic accidents (Galéra et al., 2012) and medical malpractice (Smallwood et al., 2011b). If the latter seems counterintuitive, it has to be considered that MW has been shown to affect even higher processes such as decision making, for example by making choices more likely to be biased by past experiences (Demanet et al., 2013).

With regards to the effects on health and wellbeing, Killingsworth and Gilbert (2010) suggested that MW predicts daily unhappiness, whereas other studies support the opposite pathways with negative mood being the cause of an increased tendency of the mind to wander (Smallwood et al., 2009b; Smallwood and O'Connor, 2011; Stawarczyk et al., 2013). Moreover, MW has been associated with the occurrence of psychopathological disorders, such as dysphoria (Smallwood et al., 2007b; Carriere et al., 2008) and attention deficit hyperactivity disorder (Liddle et al., 2011). As to psychophysiological reactivity, Smallwood et al. (2004a) found heart rate (HR) accelerations during periods of MW in a sustained attention task. Similarly, Smallwood et al. (2004b) showed a positive correlation between physiological arousal (HR) and frequency of MW episodes during a semantic encoding task, and these findings were replicated in a dysphoric population (Smallwood et al., 2007b). Smilek et al. (2010) compared blink rates during probe-caught episodes of MW and on-task periods of reading, demonstrating enhancement of the blink reflex during the first condition. The supposed adverse consequences of MW on health extend to the point that this process has been associated with shorter telomere length, indicating a more rapidly aging body (Epel et al., 2013).

To our knowledge, no longitudinal studies investigated the costs of MW to health and wellbeing. This study represents a first attempt to do so by the use of multiple measures of MW in the lab and naturalistically. As measuring MW is intrinsically complicated and has been done mostly in the laboratory, which can limit spontaneity of behavior, our first specific aim was to test if the occurrence of episodes of MW in the lab would predict MW in everyday life 1 year later. We wanted to study if: (a) laboratory assessments of MW have ecological validity and (b) whether the tendency to MW is a stable characteristic of the individual. Second, we examined if the likelihood of being a “mind wanderer” was associated with specific personality dispositions. Third, we tested if the frequency of MW longitudinally acted as a protective or a risk factor for health, accounting for the effects of biobehavioral variables. As high ambulatory HR and its variability (HRV) have been shown to predict total and non-cardiovascular mortality (reviewed in Hansen et al., 2008 and Thayer et al., 2010), these two variables were used as indices of health vulnerability. Somatic symptoms at follow up were considered as another measure of health vulnerability, in light of the association between somatization tendencies and repetitive thinking (Verkuil et al., 2010) and given that MW has been considered a form of repetitive thinking (reviewed in Watkins, 2008). It has to be noted that among various types of repetitive thought, basic research mainly focused on MW, whereas clinical research had a long lasting interest in rumination and worry, highlighting their role in the onset and maintenance of psychopathology (Aldao et al., 2010). Most researchers, however, included rumination and worry in their operationalization of MW, making it difficult to disentangle the effect of MW *per se*. As the aim of this study was to clarify the effects of non-pathological MW, the latter was assessed independently from rumination and worry both at state and trait levels. Indeed, there is evidence that repetitive thinking such as rumination and worry also predict longer sleep latency (e.g., Zoccola et al., 2009), but no studies have examined this association in the case of MW. It seems intuitively plausible that MW would have a disturbing effect during the same phase of the sleep process, that is, when falling asleep, therefore this specific sleep difficulty was assessed as our last marker of physical and psychological health (e.g., Taylor et al., 2003).

## Materials and methods

### Participants

Seventy-three subjects participated in the laboratory session of the study (described in Ottaviani et al., 2013) and 45 agreed to be contacted at follow up. Of these 45, one participant did not complete the ambulatory session and 3 were excluded due to excessive artifacts or inconsistent diary entries. The final sample was composed of 40 subjects [21 women, 19 men; mean age = 24.5 (4.9) years], recruited among students at Sapienza University of Rome. All subjects were Caucasian. Exclusionary criteria were: a current or past diagnosis of psychiatric disorders, diagnosis of hypertension or heart disease, use of drugs/medications that might affect cardiovascular function, obesity (body mass index >32 kg/m^2^), menopause, pregnancy, or childbirth within the last 12 months. Participants were compensated (€ 25) for their time. The protocol was approved by the Bioethical Committee of S. Lucia Foundation, Rome.

### Procedure

The study consisted of two phases: a laboratory session at time 0 and an ambulatory session at time 1. The average time between the two sessions was 13.9 (1.2) months.

At time 0, participants were informed of the following restrictions: no caffeine, alcohol, nicotine, or strenuous exercise for 2 h prior to the laboratory session. After reading and signing the informed consent form and a 5-min physiological baseline recording, participants were engaged in two 5-min recall interviews; after each interview, they performed a 20-min tracking task with thought probe. The rationale for the interviews was to increase the likelihood of episodes of MW and perseverative cognition, as the primary goal of the laboratory session was to study the psychophysiological correlates of these cognitive states. Detailed findings from the laboratory session are besides the scope of this study and have been described elsewhere (Ottaviani et al., 2013). The first interview required participants to verbally describe a well-known route (i.e., the itinerary from the building where the experiment took place to Rome central station), while, in the second interview, participants were asked to talk about a negative personal event that occurred in the past or will occur in the future and that would elicit stress/worry when “when thinking about it.” At the end of the tasks, participants completed a series of on line personality questionnaires.

At follow up, appointments were scheduled by e-mail. During their visit to the lab, participants were instructed about the use of the electronic diary and the ambulatory HR device. The belt was attached, and the participants left the laboratory. The next morning, they were asked to return the diary and apparatus to the laboratory, were debriefed, and received monetary compensation.

### Tracking task with thought probe (t0)

Only measures that are relevant to the aims of the present study will be reported [see Ottaviani et al. (2013) for specific details about the task]. The task was developed using Superlab 4 software (Credus Corporation). To increase the likelihood of episodes of MW and make the task automatic, the level of difficulty was very low. Participants were asked to keep the cursor inside a white circle in motion on a black screen and press the left mouse button as fast as possible each time the circle turned red. At different time intervals, probes interrupted the task to inquire about subjects' thoughts. The thought probe method used in this study was adapted from Stawarczyk et al. (2011). We had a total of 16 thought-probes per subject (8 during each tracking task). For each probe, participants were asked to characterize the ongoing conscious experience they had just prior to the probe, among the following: (a) focused on the task, (b) distracted by external stimuli (noise, etc.), (c) MW, (d) worrying about a future event, (e) ruminating about a past stressful event. The only variable that was analyzed in the present study was the number of episodes of MW during the two 20-min tracking tasks (aggregated).

### Psychophysiological assessment (t0)

The electrocardiogram (ECG) was continuously monitored (Monitoring, Adatec s.r.l., Italy) with a standard electrode configuration. The signal was digitized at 1000 Hz. Each epoch was manually checked and corrected for artifacts. HR and the root mean square of successive differences (RMSSD), which primarily reflects vagally mediated HRV, were derived using Kubios HRV Analysis Software (Niskanen et al., 2004). HR and HRV relative to the 5-min baseline recorded before the beginning of the first interview were used as predictors in the regression analyses.

### Questionnaires (t0)

At time 0, participants completed on line a series of socio-demographic (age and sex), lifestyle (nicotine, alcohol, and caffeine consumption, physical exercise), and personality scales: (a) Stress-Reactive Rumination Scale (SRRS; Robinson and Alloy, 2003), (b) Penn State Worry Questionnaire (PSWQ; Meyer et al., 1990), (c) State-Trait Anxiety Inventory (STAI-X2; Spielberger et al., 1970), (d) Center for Epidemiologic Studies Depression Scale (CES-D; Radloff, 1977), (e) Five Facets Mindfulness Questionnaire (FFMQ; Baer et al., 2006), (f) somatization subscale of the Symptom Checklist-90 Revised (SCL-90 R; Derogatis, 1977), (g) PROMIS Sleep Disturbance-short form (Yu et al., 2011). The SSRS requires to indicate how frequently one would engage in a series of activities (e.g., “Think about how the negative event will negatively affect your future”) in response to a stressful event on a scale from 0 (Not focus on this at all) to 100 (Focus on this to a great extent). As only the Negative Inferential Style (NIS) subscale has been previously associated with ruminative tendencies (Robinson and Alloy, 2003), data related to this subscale of the SRRS were analyzed in the present study. The PSWQ is a 16-item self-report questionnaire commonly used to measure the tendency to worry in an excessive and uncontrollable way (e.g., “Once I start worrying, I cannot stop”) on a on a 5-point scale ranging from 0 (Not at all) to 4 (Most of the time). The STAI—X2 consists of 20 items with multiple choice answers (never, sometimes, often, and always) directed at investigating relatively stable individual differences in trait anxiety. The CES-D is a 20-item self-report scale that assesses the frequency of occurrence of symptoms of depression during the past week. The FFMQ assesses five facets of a general tendency to be mindful in daily life (observing, describing, acting with awareness, non-reactivity to inner experience, and non-judging of inner experience) on a 5-point Likert scale ranging from 1 (never or very rarely true) to 5 (very often or always true). The SCL-90-R somatization subscale is a 12-item measure of commonly experienced physical symptoms that has been widely used as a standalone index of somatization severity (e.g., Güleç et al., 2013). The PROMIS Sleep Disturbance-Short form is an 8-item scale that has been shown to be useful for grading the global severity of insomnia (Yu et al., 2011); the present study focused on scores of the item “I had difficulties falling asleep.”

### Ambulatory session (t1)

HR was recorded as beat-to-beat intervals using a t6 Suunto Memory Belt (SuuntoVantaa, Finland), sampling at 1000 Hz. The Suunto Memory Belt has been shown to be a reliable device to measure the ECG compared to a 5-lead ECG (Weippert et al., 2010). Participants were asked to return the HR recorder after 24-h of wearing or in case of any difficulties. Raw beat-to-beat intervals (IBI) were analyzed according to the Task Force Guidelines (1996). The 24-h IBI data were decomposed into 5-min blocks. Each epoch was manually checked and corrected for artifacts. The Kubios HRV Analysis Software (Niskanen et al., 2004) was used to calculate the HRV time domain parameter (RMSSD). After excluding blocks with more than 5% artifact rate, we calculated the average beats per minutes (HR), and RMSSD (HRV). Twenty-four hour HR and HRV were used in the analyses.

### Electronic diary (t1)

Participants were provided with an electronic diary implemented on an Android phone via the SurveyPocket App (Questionpro.com). At random times (about every 30 min), the phone signaled participants that it was time to report the specific ongoing cognitive process (focused on the task, distracted by external stimuli, MW, worrying about a future event, ruminating about a past stressful event) and information on factors that may affect HR, including posture, physical activity, and food, caffeine, nicotine, and alcohol consumption since the last diary report. The other questions in the diary are not relevant for the aims of the present study and will not be described here. Before bedtime, subjects were asked to fill out the Patient Health Questionnaire [PHQ-15 for somatization Kroenke et al. (2002)] and, upon awakening, the PROMIS Sleep Disturbance-Short Form, both implemented on the same Android phone.

### Statistical analyses

Data are expressed as means (SD). To correct for multiple comparisons, only Bonferroni adjusted *p*-values are presented. Laboratory data processing and data analyses were performed with Systat 11.0 (Systat Software Inc., Richmond, CA).

The effects of biobehavioral (body mass index, age, years of education, physical activity, alcohol, nicotine consumption) and personality factors (scores at the STAI, CES-D, SRRS, PWSQ, and FFMQ) on the dependent variables (24-h HR and HRV, somatization levels, and difficulties falling asleep after 1 year) were analyzed by Pearson correlations. Differences due to sex were analyzed by *t* test.

To test if laboratory assessments of MW have ecological validity and the tendency to MW is a stable characteristic of the individual, we first ran Pearson correlations between frequency of episodes of MW in the lab and in daily life (1 year later). Second, we examined if the likelihood of being a “mind wanderer” was associated with specific personality dispositions. To do so, Pearson correlations between the frequency of episodes of MW and scores of the dispositional questionnaires (STAI, CES-D, SRRS, PWSQ, and FFMQ) were computed. Third, we tested if the frequency of MW longitudinally acted as a protective or a risk factor for health, accounting for the effects of biobehavioral variables. A series of multiple regression analysis were conducted according to the following model:

$$Y=a+B_{1}X_{1}+B_{2}X_{2}+B_{3}X_{3}+B_{4}X_{4}+B_{5}X_{5}$$

where (1) a (Alpha) is the constant or intercept; (2) *Y* is each examined dependent variable (24-h HR, 24-h HRV, scores of the PHQ-15, and scores of the item “I had difficulty falling asleep” of the PROMIS sleep scale, respectively), and (3) *X*_1_, *X*_2_, *X*_3_, *X*_4_, and *X*_5_ are the predictors for that specific model (e.g., sex, baseline level of the dependent variable at time 0, occurrence of episodes of MW at time 0, and occurrence of episodes of MW at time 1). Results are reported in terms of both the regression coefficients (B) and the standardized regression coefficients (β), which are obtained by applying the regression models to standardized dependent and independent variables. Statistical significance of the standardized coefficients was tested by *F*-tests.

To control for the effects of biobehavioral variables without decreasing too much the degrees of freedom for the present sample size, only those that had a significant bivariate correlation with a given dependent variable were entered in the subsequent regression models.

## Results

The only significant associations that emerged between socio-demographic variables and our outcome measures were: (1) nicotine consumption and 24-h HR (*r* = 0.33; *p* = 0.04), (2) worry tendencies (PSWQ) and 24-h HRV (*r* = −0.42; *p* = 0.01), (3) ruminative tendencies (NIS) and somatization (*r* = 0.33; *p* = 0.04), and (4) trait anxiety (STAI) and difficulties falling asleep (*r* = 0.36; *p* = 0.02), thus these variables were included as predictors in the corresponding multiple regression models.

Table 1 shows sex differences for the main variables of the study. The only significant difference regarded higher levels of depressive symptoms in women compared to men [*t*_(38)_ = 2.1, *p* = 0.04] and higher baseline HR in men compared to women at baseline [*t*_(38)_ = −2.2, *p* = 0.04], therefore sex was included as a predictor in all the multiple regression models.

**Table 1:** **Sex differences for the main variables of the study**.

	**Women (n = 21)**	**Men (n = 19)**	***p***
**TIME 0**			
Age (years)	23.3 (5.3)	25.8 (4.2)	0.11
Body Mass Index (Kg/m^2^)	22.3 (6.5)	23.2 (2.5)	0.63
Baseline HR	77.9 (13.1)	87.7 (15.4)	0.04^*^
Baseline HRV	40.1 (11.4)	34.9 (8.9)	0.12
MW episodes (*n*)	4.8 (3.4)	3.9 (2.1)	0.34
CES-D	18.5 (10.2)	12 (9.1)	0.04^*^
STAI	43.7 (9.1)	41.5 (5.9)	0.37
PSWQ	45.1 (13.6)	42.8 (11.7)	0.57
Negative Inferential Style (SRRS)	41.1 (15.9)	39.2 (11.8)	0.66
Observing (FFMQ)	23.3 (4.1)	22.5 (5.1)	0.58
Describing (FFMQ)	23.8 (3.0)	21.7 (3.7)	0.06
Awareness (FFMQ)	25.9 (3.9)	24.4 (3.3)	0.19
Non-judging (FFMQ)	23.0 (4.2)	22.4 (4.5)	0.65
Non-reactivity (FFMQ)	19.9 (3.3)	20.9 (3.7)	0.40
Difficulties falling asleep (PROMIS)	3.0 (1.3)	2.6 (1.2)	0.29
SCL-90 R (somatization)	20.1 (8.0)	17.1 (6.0)	0.19
**TIME 1 (1 YEAR)**			
MW episodes (*n*)	6.1 (3.5)	7.7 (4.1)	0.19
24-h HR (bpm)	72.3 (9.2)	74.2 (7.1)	0.43
24-h HRV (ms)	25.7 (5.9)	24.6 (5.5)	0.64
Difficulties falling asleep (PROMIS)	2.0 (1.4)	2.9 (1.4)	0.06
PHQ-15	17.8 (4.6)	17.9 (5.1)	0.95

* p < 0.05.

**Figure 1:**
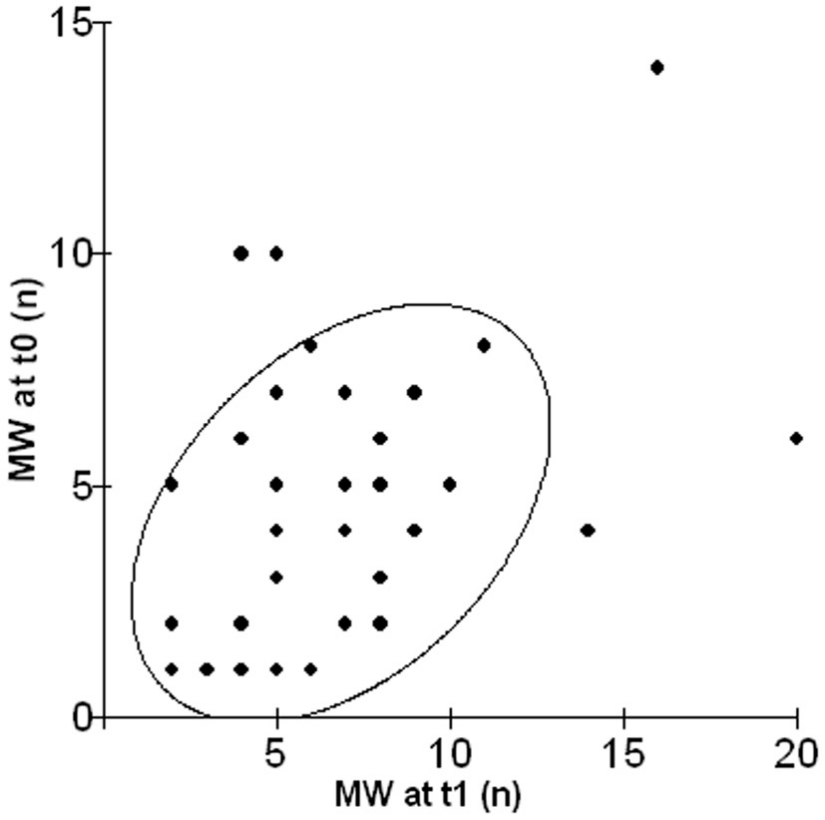
**Scatterplot illustrating the relationship between the number of episodes of MW in the lab (MW at t0) and in daily life after 1 year (MW at t1)**.

As shown in Figure 1, a significant relationship emerged between frequency of episodes of MW in the lab and in daily life after 1 year (*r* = 0.41, *p* = 0.01).

**Figure 2:**
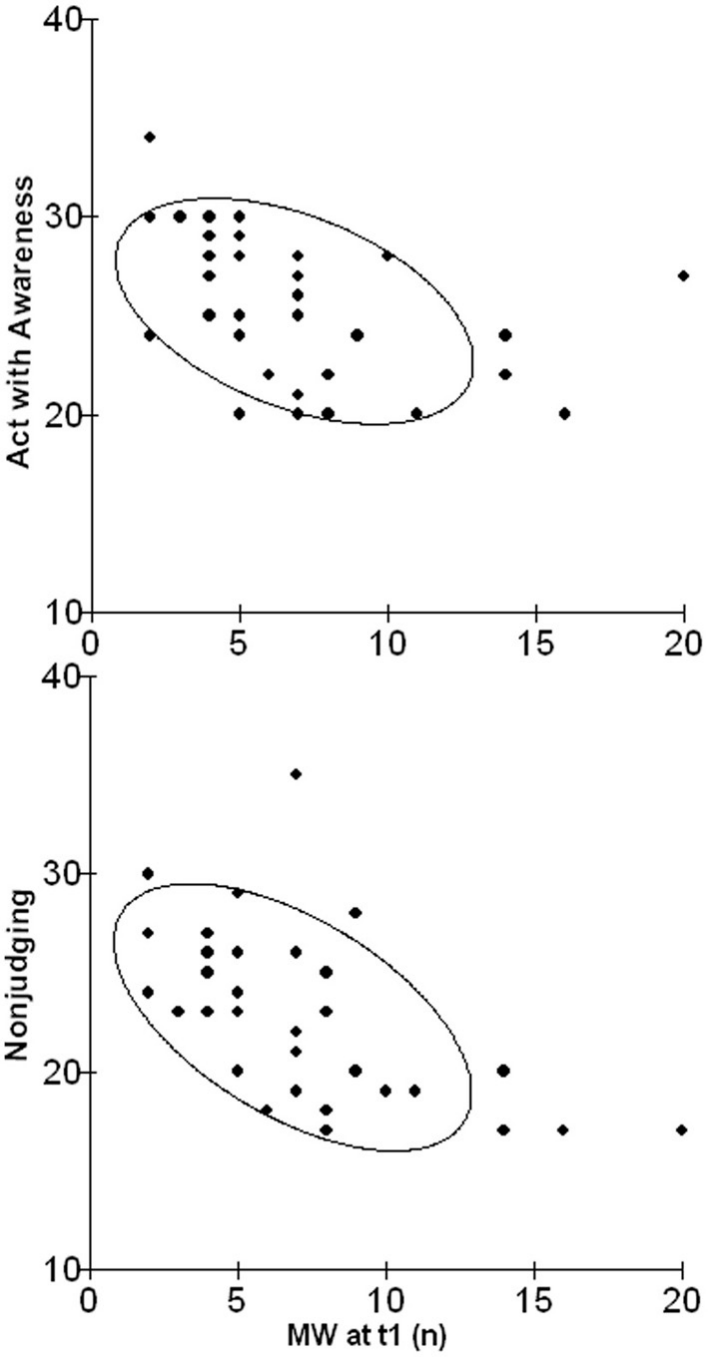
**Scatterplots illustrating the relationship between scores of the subscale Acting with Awareness (upper graph) and Non-judging (lower graph) of the FFMQ and the occurrence of episodes of MW in daily life after 1 year**.

As to personality dispositions, a relationship between scores of the subscales Non-judging and Acting with awareness of the FFMQ were significantly related with the occurrence of MW episodes in daily life (*r* = − 0.56, *p* = 0.001 and *r* = − 0.42, *p* = 0.03; scatterplots depicted in Figure 2).

With regard to the prediction of psychophysiological risk factors for health, Table 2 shows the results of the multiple regressions for 24-h HR and 24-h HRV. Model 1 accounted for 30% of the variance of 24-h HR, with a significant effect of nicotine consumption (*F* = 5.86; *p* = 0.02) and MW at time 1 (*F* = 7.26; *p* = 0.01) as predictors. In Model 2, baseline HRV at time 0 (*F* = 31.52; *p* < 0.0001) and trait worry (*F* = 5.05; *p* = 0.03) were significant predictors of 24-h HRV, accounting for 63% of the variance.

**Table 2:** **Summary of multiple regression analysis for the prediction of 24-h HR (Model 1) and 24-h HRV (Model 2) at time 1**.

**Model 1 (24-h HR)**				**Model 2 (24-h HRV)**			
	**B**	**SE**	**β**		**B**	**SE**	**β**
Sex	1.67	1.32	0.21	Sex	0.33	0.63	0.06
Baseline HR (t0)	0.03	0.10	0.06	Baseline HRV (t0)	0.38	0.07	0.71^**^
Smoking	0.87	0.36	0.38^*^	PSWQ	−0.12	0.05	−0.26^*^
MW (t0, lab)	−0.27	0.47	−0.10	MW (t0, lab)	0.00	0.23	0.00
MW (t1, EMA)	0.96	0.36	0.46^*^	MW (t1, EMA)	−0.26	0.19	−0.18
*R*^2^		0.30		*R*^2^		0.63	

B, unstandardized regression coefficient; SE, standard error of the regression coefficient; β, standardized regression coefficient.

* p < 0.05;

** p < 0.0001.

Table 3 shows the multiple regression models for the prediction of self-reported risk factors for health at time 1. Trait rumination and somatization tendencies at time 0 (SCL-90 R) were significant predictors of somatization at time 1 (*F* = 4.56; *p* = 0.04 and *F* = 30.3; *p* < 0.0001, respectively). Specifically 54% of the variance of the PHQ-15 was accounted for by Model 3. In Model 4, gender (*F* = 4.37; *p* = 0.04) and MW at time 1 (*F* = 4.97; *p* = 0.03) were significant predictors of difficulties of falling asleep at time 1, accounting for 42% of the variance.

**Table 3:** **Summary of multiple regression analysis for the prediction of somatization tendencies (Model 3) and difficulties falling asleep (Model 4) at time 1**.

**Model 3 (PHQ-15)**				**Model 4 (difficulties falling asleep)**			
	**B**	**SE**	**β**		**B**	**SE**	**β**
Sex	−1.01	0.61	−0.21	Sex	−0.43	0.21	−0.30^*^
Somatization (t0)	0.43	0.08	0.65^**^	Baseline (t0)	0.29	0.16	0.25
NIS	0.09	0.04	0.25^*^	STAI	0.04	0.03	0.20
MW (t0, lab)	0.30	0.22	0.18	MW (t0, lab)	0.02	0.07	0.05
MW (t1, EMA)	0.00	0.17	0.00	MW (t1, EMA)	0.13	0.06	0.35^*^
*R*^2^		0.54		*R*^2^		0.42	

B, unstandardized regression coefficient; SE, standard error of the regression coefficient; β, standardized regression coefficient.

* p < 0.05;

** p < 0.0001.

## Discussion

The present study was designed to prospectively examine three characteristics of MW: its stability over time, its relationship with determined personality measures, and its role as a predictor of established risk factors for health.

First, a surprisingly high correlation emerged between the frequency of episodes of MW at time 0 and the same measure at time 1. This result is particularly relevant if we consider that the two assessments took place not only at different times (about 1 year apart) but also in totally different contexts (i.e., in the laboratory and in participant's daily life). The stability of MW across different contexts had already been studied by McVay et al. (2009) with consistent results: subjects who reported more MW during a laboratory task endorsed more MW experiences during everyday life. Similarly, Unsworth et al. (2012) measured various cognitive abilities in the laboratory and then recorded everyday attention failures, such as MW or distraction in a diary over the course of a week, supporting evidence for the ecological validity of laboratory measures of attention control. Our study replicated and extended these findings, with the introduction of the longitudinal dimension between the two measures of MW.

With regard to dispositional variables, although the role of MW as a marker for depressive thinking had been previously highlighted, as shown by studies linking this cognitive process to dysphoria (Smallwood et al., 2007b; Carriere et al., 2008) and negative moods (Smallwood et al., 2009b; Killingsworth and Gilbert, 2010; Smallwood and O'Connor, 2011; Stawarczyk et al., 2013), we failed to replicate an association between the occurrence of MW and depressive symptoms both cross-sectionally and longitudinally. A possible explanation for the inconsistency may derive from the fact that previous studies included ruminative thoughts in their conceptualization of MW. Although MW, rumination, and worry are often included under the same umbrella term of “repetitive thinking,” the only study that directly compared these processes in terms of their affective correlates, suggested that the negative effects of MW on moods vanish when differentiated from perseverative cognition (Ottaviani et al., 2013). In agreement with previous results, a negative association between measures of dispositional mindfulness and MW emerged. The reciprocal link between these two apparently opposite constructs has been recently confirmed by Mrazek and colleagues (Mrazek et al., 2012), who found an inverse relationship between a dispositional measure of mindfulness (i.e., the Mindful Attention and Awareness Scale) and converging measures of both self-reported and indirect markers of MW. Here, we replicated these results by using a different self-report measure, i.e., the FFMQ that further allowed us to provide insights on which specific facets of mindfulness would be more closely linked to MW tendencies. The non-judging and acting with awareness features emerged as the most relevant, again with surprisingly strong correlations. This constitutes an intriguing result as these are the two facets that play the most important role in mindfulness clinical applications, such as Mindfulness Based Stress Reduction (MBSR) and Mindfulness Based Cognitive Therapy (see Grossman et al., 2004; Chiesa and Serretti, 2009 for reviews). Indeed they are the two most effective factors in preventing intrusive thoughts: increased awareness may allow patients to break the ruminative cycle by attending to the present and not to the past, and non-judging may foster acceptance rather than avoidance and its ironic effects on unwanted thoughts (“white bear effect,” Wegner et al., 1987).

As to the examined risk factors for health, MW appeared to be associated with short term maladaptive consequences but not with noxious effects 1 year later. In fact, MW at time 0 was not a significant predictor in any of the regression models, while the frequency of episodes of MW during the ecological momentary assessment predicted 24-h HR in the same day and difficulties falling asleep the subsequent night. Results on the association between MW and simultaneous increases in HR had been already demonstrated by Smallwood et al. (2004a,b, 2007b) during a series of laboratory task and were here replicated in a more ecological setting. Surprisingly, no previous studies investigated the relationship between MW and sleep difficulties. However, on the flip side, evidence suggests that increased practice of mindfulness techniques is associated with improved sleep and that MBSR participants experience a decrease in sleep-interfering cognitive processes (reviewed in Winbush et al., 2007). Taken together, findings argue for a link between MW and sleep difficulties that needs to be further investigated.

Interestingly, baseline HRV and worry tendencies, assessed by the PSWQ, were significant predictors of 24-h HRV 1 year later and the amount of variance the model accounted for was particularly large (63%). The stability of HRV over time has been extensively demonstrated (e.g., Bertsch et al., 2012). As worry was significant in the prediction but MW was not, it seems evident that a distinction needs to be made between future-oriented MW, which has been associated with autobiographical planning (Baird et al., 2011; Smallwood et al., 2011a) and future worrisome thoughts, which have conversely been related to decreased HRV both during the day and the subsequent night (Brosschot et al., 2007; Pieper et al., 2010).

Finally, trait rumination and somatization tendencies at time 0 significantly predicted somatization at time 1. Again, this results fits with a large amount of data linking perseverative cognition with somatization tendencies (see Verkuil et al., 2010 for a review). Still, it seems that MW refers to a different phenomenon, which is less pathogenic as it is probably not associated with the sustained physiological reactivity that has been shown during rumination (e.g., Ottaviani et al., 2009, 2011; Ottaviani and Shapiro, 2011). Although being the first prospective study in the field, the fact that we did not find maladaptive consequences of MW in the long term is not a standalone result. There are studies showing greater life satisfaction and socio-emotional well-being associated with a particular form of MW, that is daydreaming about close family and friends (Mar et al., 2012).

This study has several limitations. First, the sample size was relatively small and may not have been adequate in some of the comparisons. Second, MW was treated as a dichotomy and measured using self-reports, whereas it has recently been demonstrated that it is possible to be mindless at different degrees (Schad et al., 2012). Also, we examined 24-h HR and HRV without synchronizing the occurrence of episodes of MW with psychophysiological recordings. We did so, as we were more interested on established risk factors for health and not on the physiological correlates of MW. Finally, our sample was composed of healthy students and 1 year may not be enough to see the long term maladaptive consequences of MW in terms of effects on health risk factors.

Limitations notwithstanding, our preliminary findings extend the results of previous studies by showing MW to be a relatively stable characteristic of the individual, inversely related to specific mindfulness facets such as acting with awareness and non-judging and to have short term negative effects on health and wellbeing (24-h HR and difficulties falling asleep). However, the data failed to show any long term pathogenic effects of MW. Results emphasize the need of prospective studies to clarify under which circumstances the so common process of MW takes the form of pathological rumination or worry, with clear implications for both prevention and therapy.

### Conflict of interest statement

The authors declare that the research was conducted in the absence of any commercial or financial relationships that could be construed as a potential conflict of interest.
